# Evaluation of testicular shielding effectiveness for surface and internal photon dose using a 3D‐printed scrotal phantom

**DOI:** 10.1002/acm2.70842

**Published:** 2026-10-05

**Authors:** Joseph Speth, Josh Kilian‐Meneghin, Salma K. Jabbour, Yin Zhang, Xiao Wang, Zhenyu Xiong, Ke Nie, Ning J. Yue, Xinxin Zhang

**Affiliations:** ^1^ Department of Radiation Oncology Rutgers Cancer Institute of New Jersey, Rutgers Robert Wood Johnson Medical School New Brunswick New Jersey USA; ^2^ Department of Radiation Oncology Robert Wood Johnson Barnabas Health Livingston New Jersey USA

**Keywords:** out‐of‐field dose, radiation therpay, testicular shielding

## Abstract

**Background:**

Testicular shielding has long been used to reduce testicular dose during radiation treatment. However, quantitative evaluations of the effectiveness of such shielding in the literature vary substantially.

**Purpose:**

In this study, the dose reduction expected at both the testicular skin surface and the inside of the testes was evaluated under different irradiation conditions, to enable informed clinical decision‐making.

**Methods:**

A 3D printed scrotal phantom was designed to simulate patient testes and was composed of three layers to enable dose measurement inside the phantom. Gafchromic EBT3 film was used for dose measurements. The scrotal phantom was attached to a RANDO anthropomorphic phantom for computed tomography scanning and measurements. A commercial testicular shield with a lead wall thickness of 1.27 cm and a diameter of 6 cm was used for shielded measurements. The measured absolute dose was normalized to the delivered MU for each measurement to obtain a dose‐to‐MU value (cGy/MU). Both dose‐to‐MU values and shielding effectiveness were evaluated with a stationary anterior‐posterior beam at different distances from field edge to scrotal phantom. In addition, the energy dependence of shielding effectiveness was assessed using two volumetric modulated arc therapy (VMAT) plans with identical parameters and modulation but using 6 and 15 MV energies.

**Results:**

The shielded and unshielded dose‐to‐MU values generally decreased with increasing field‐to‐phantom distance for both surface and internal dose measurements. However, the shielding ratio remained relatively constant across distances, with a reduction factor of approximately 3.3 for surface dose and 2.7 for internal dose. Minimal energy dependence was observed in both internal and surface dose measurements, as well as in shielding effectiveness, across the two VMAT plans. The overall shielding ratio was approximately 2.3 for both plans.

**Conclusions:**

Testicular shielding reduced both surface and internal testicular dose by approximately a factor of 2 to 3, demonstrating consistent effectiveness across different irradiation geometries and photon energies.

## 1 INTRODUCTION

Radiotherapy is often used to treat malignancies near the testes. Although the testes are typically outside the primary treatment field, head leakage and patient scatter can still result in a non‐negligible dose. Testicular dose as low as 0.2 Gray (Gy) has been associated with transient reduction in sperm count.^1^, ^2^, ^3^ The dose range of approximately 0.2 to 0.8 Gy has been associated with temporary oligospermia,^1^, ^2^, ^4^, ^5^ and doses beyond about 0.8 Gy have been found to cause transient azoospermia.^1^, ^5^ Sperm count reduction and testicular functional impairment increase in both severity and duration with dose and fractionated delivery.^1^, ^3^, ^5^, ^6^ Sandeman found that the threshold for permanent azoospermia dropped as low as 2.5 Gy when administration was fractionated, compared to a threshold of around 6 Gy for the same effect from a single dose.^6^ Therefore, testicular shielding may be clinically warranted to reduce dose to the testes.

Published studies have reported widely varying shielding effectiveness, with reported reductions ranging from approximately 10% to over 90% depending on treatment conditions and measurement approach (Table S1).^7^, ^8^, ^9^, ^10^, ^11^, ^12^, ^13^, ^14^, ^15^ Lieng, et al. reported a reduction of testicular dose from 28.6 to 2.6 cGy with use of a 1.25 cm thick lead shield and MOSFET detectors,^7^ while Fricker measured scatter dose reduction of 10% using a custom‐made lead testicular shield and radiochromic film.^8^ When expressed relative to prescribed dose, testicular dose has been reported to range from about 0.07% to 1.7–1.8% across different clinical scenarios.^7^, ^13^, ^16^ In addition, the dependence of shielding effectiveness on testicular distance from the treatment field is inconsistent. Fraass, et al. observed greater shielding effectiveness at shorter distances from treatment fields using custom lead shields.^9^ Singhal, et al. and Ravichandran, et al. used similar designs of low melting point alloy custom shielding; however, their findings diverge. Singhal, et al. reported an increase in shielding ratio with distance, from 7.22 at 3.5 cm to 10.33 at 8 cm,^15^ whereas Ravichandran et al. observed minimal dependence on distance.^14^

Previous studies of testicular shielding have generally reported either surface or internal dose, limiting direct comparison between these measurements and potentially contributing to the variability described above. In this study, dose was measured at both the surface and within a testicular phantom using radiochromic film, enabling evaluation of shielding effectiveness at both locations. The dependences of shielding ratio on distance between field and testicle, as well as on beam energy, were also assessed with three‐dimensional conformal radiotherapy (3DCRT) and VMAT.

## 2 METHODS

### 2.1 Scrotal phantom

A spherical scrotal phantom was designed to hold EBT3 Gafchromic film (Ashland Inc., Bridgewater, NJ) within a commercially available lead (Pb) testicular shield (Radiation Products Design, Inc., Albertville, MN) with an inner diameter of approximately 6.35 cm and a Pb wall thickness of 1.27 cm, which is the size commonly used at our institution. All measurements were performed using this shield configuration. The phantom was produced using an Ultimaker S5 3D printer (Ultimaker B.V., Utrecht, The Netherlands) equipped with a 0.4 mm nozzle. The phantom was sectioned along two parallel planes, each offset by 5 mm from the central plane of the sphere, allowing film dosimetry to be performed within the phantom (Figure 1a). This phantom was designed with an external diameter of 5.9 cm, allowing placement of film between the phantom and the testicular shield while accommodating potential printer geometric inaccuracies. The phantom was modeled in TinkerCAD (Autodesk Inc, San Francisco, CA) and exported as an STL file to the Ultimaker Cura software package, which generated the printer instruction set for the Ultimaker S5 printer. The printer used polylactic acid (PLA) filament with 100% infill in a lattice pattern. PLA was selected because its nominal specific gravity of 1.24 (Ultimaker data sheet) is closer to that of water than other available printing materials, allowing the phantom to approximate soft tissue density for scatter dose measurement. The homogeneity and dimensions of the phantom were verified using computed tomography (CT) images acquired with 2.5 mm slice thickness on a GE RT16 Lightspeed CT scanner (GE Healthcare, Waukesha, WI) and the average CT number of the phantom was about ‐150 HU.

**FIGURE 1:**
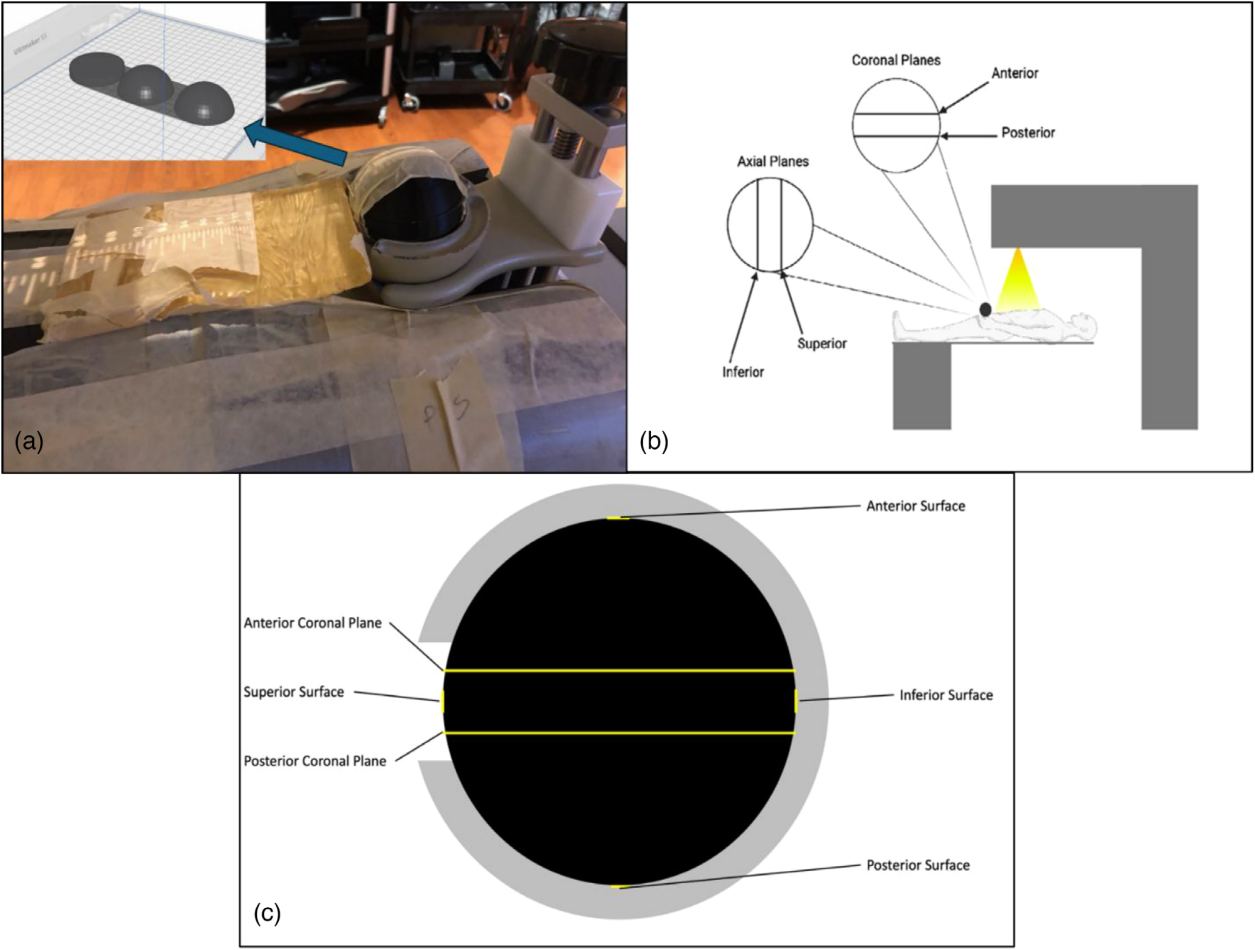
Setup for measurement with testicle shield and a virtual representation of the three scrotal phantom sections in Ultimaker S5 software (Figure 1a); Internal dose plane geometry and nomenclature for two orientations of the scrotal phantom (Figure 1b, produced using BioRender, Canada); Cross sectional view of film placement within the testicular shield (Figure 1c).

### 2.2 Data collection

Phantom irradiation was performed using Varian TrueBeam linear accelerators (Varian Medical Systems, Palo Alto, CA). All modulated fields were delivered on the machine equipped with a high definition multileaf collimator with leaf widths of 2.5 and 5 mm. The head leakage measured at 1 m away from isocenter in horizontal plane was approximately 0.02%. The scrotal phantom was used in conjunction with a RANDO anthropomorphic pelvis phantom (Radiology Support Devices, United Kingdom) to simulate a head‐first supine patient geometry. Figure 1a shows the setup for dose measurement, where a superflab bolus was placed between the scrotal and RANDO phantoms to minimize the intervening air gap.

To evaluate the effectiveness of the testicular shielding as a function of distance from the treatment field edge to the scrotal phantom (field‐to‐phantom distance), a 6 MV photon beam with a 10 × 10 cm^2^ field size was delivered at a 100 cm source‐to‐surface distance in the anterior‐posterior direction. The phantom assembly was moved longitudinally along with the treatment couch to vary the field‐to‐phantom distance. Measurements were obtained at five distances (1, 2, 3, 5, and 8 cm). To maintain adequate film signal‐to‐noise ratio at low out‐of‐field dose levels, monitor units (MU) were increased at greater distances and when the testicular shield was used. The MUs ranged from 6000 to 10 000 for unshielded dose measurements and from 18 000 to 35 000 for shielded dose measurements. All the measured doses were normalized to the delivered MUs (dose‐to‐MU ratio). The shielding ratio was defined as the ratio of dose‐to‐MU measured without shielding to that measured with shielding in an otherwise identical setup.

To simulate clinical rectal treatment and evaluate the effect of beam energy on shielding efficacy, two VMAT treatment plans were generated in the Eclipse V16.01 treatment planning system (Varian Medical Systems, Palo Alto, CA). Beam energies of 6 and 15 MV were used and the prescribed dose was 50 Gy in 10 fractions. The target volume and surrounding organs‐at‐risk were contoured on the CT images of the RANDO pelvis phantom with the scrotal phantom in place. The 6 MV VMAT plan was optimized to conformally treat a target volume approximating a clinical rectal planning target volume. The shortest distance from the target volume to the scrotal phantom was approximately 3 cm, ensuring that no primary beam entry or exit occurred through the scrotal phantom. The 15 MV plan was generated by preserving all beam parameters from the 6 MV plan and modifying only the beam energy, allowing the effect of photon energy on shielding efficacy to be evaluated while minimizing confounding factors such as differences in plan modulation. The total MUs per fraction were 2123.9 for both plans. Each plan was delivered 35 times for the shielded measurements and 15 times for the unshielded measurements.

For all measurements, a single batch of Gafchromic EBT3 film was used to measure surface and internal doses in the scrotal phantom. Surface dose measurements used rectangular film pieces approximately 1 × 2 cm^2^ in size, while internal dose measurements used circular films matching the diameter of the scrotal phantom section cuts. Each film was marked to preserve the correct orientation during scanning. Surface doses were measured at four locations (anterior, posterior, superior, and inferior) defined according to the anatomical axes of a head‐first supine patient. Internal doses were initially measured in both the axial (parallel to the beam direction) and coronal (perpendicular to the beam direction) planes at two representative field‐to‐phantom distances (1 and 2 cm) with the gantry at 0 degrees to evaluate potential plane dependence. Because only small differences were observed between the two plans at these representative locations, subsequent measurements were performed in the coronal plane (anterior and posterior) to improve measurement efficiency (Figure 1b and 1c).

### 2.3 Film calibration and statistical analysis

Dose calibration and analysis of the EBT3 film were performed using the red color channel. All films were scanned at the center of the scanner bed in the same orientation to minimize lateral scanner response non‐uniformity. Background correction was performed by subtracting pixel values of an unexposed film scanned under identical conditions (Figure S1). Each experimental condition was evaluated using a single film irradiation; therefore, experimental measurement uncertainty was not determined from repeated measurements. Based on the reported uncertainty of EBT3 film dosimetry (typically 3%–5%),^17^ an uncertainty of 5% was assumed for individual dose measurement. Propagation of this uncertainty yields an estimated relative uncertainty of approximately 7% for the calculated shielding ratios.

Statistical analyses were performed in MATLAB (Mathworks, Natick, MA) using two‐sided Wilcoxon signed‐rank tests.^18^ Distinct setup distances or measurement locations served as paired units rather than replicate measurements. Statistical significance was defined as *p*‐value < 0.05.

## 3 RESULTS

### 3.1 Surface dose variation with location and distance

Spatial variation was observed in the surface dose‐to‐MU values for both shielded and unshielded measurements. Unshielded measurements obtained at a field‐to‐phantom distance of 1 cm displayed high dose‐to‐MU values at the superior and anterior surfaces (0.07 and 0.04 cGy/MU, respectively), whereas the posterior and inferior surfaces showed lower values (0.021 and 0.015 cGy/MU, respectively). In contrast, the shielded measurements showed a different spatial distribution. The higher dose‐to‐MU values were measured at the superior (0.018 cGy/MU) and posterior (0.013 cGy/MU) surfaces, while the anterior (0.008 cGy/MU) and inferior (0.005 cGy/MU) surfaces displayed lower values. As shown in Figure 2, these patterns remained consistent across all field‐to‐phantom distances. In addition, the location‐averaged surface dose‐to‐MU trended downwards with increasing field‐to‐phantom distance, as shown in Figure S2, for both shielded (0.013 to 0.004 cGy/MU) and unshielded (0.039 to 0.011 cGy/MU) measurements. These observations suggest less contribution of scattered radiation at further distance from the treatment field.

**FIGURE 2:**
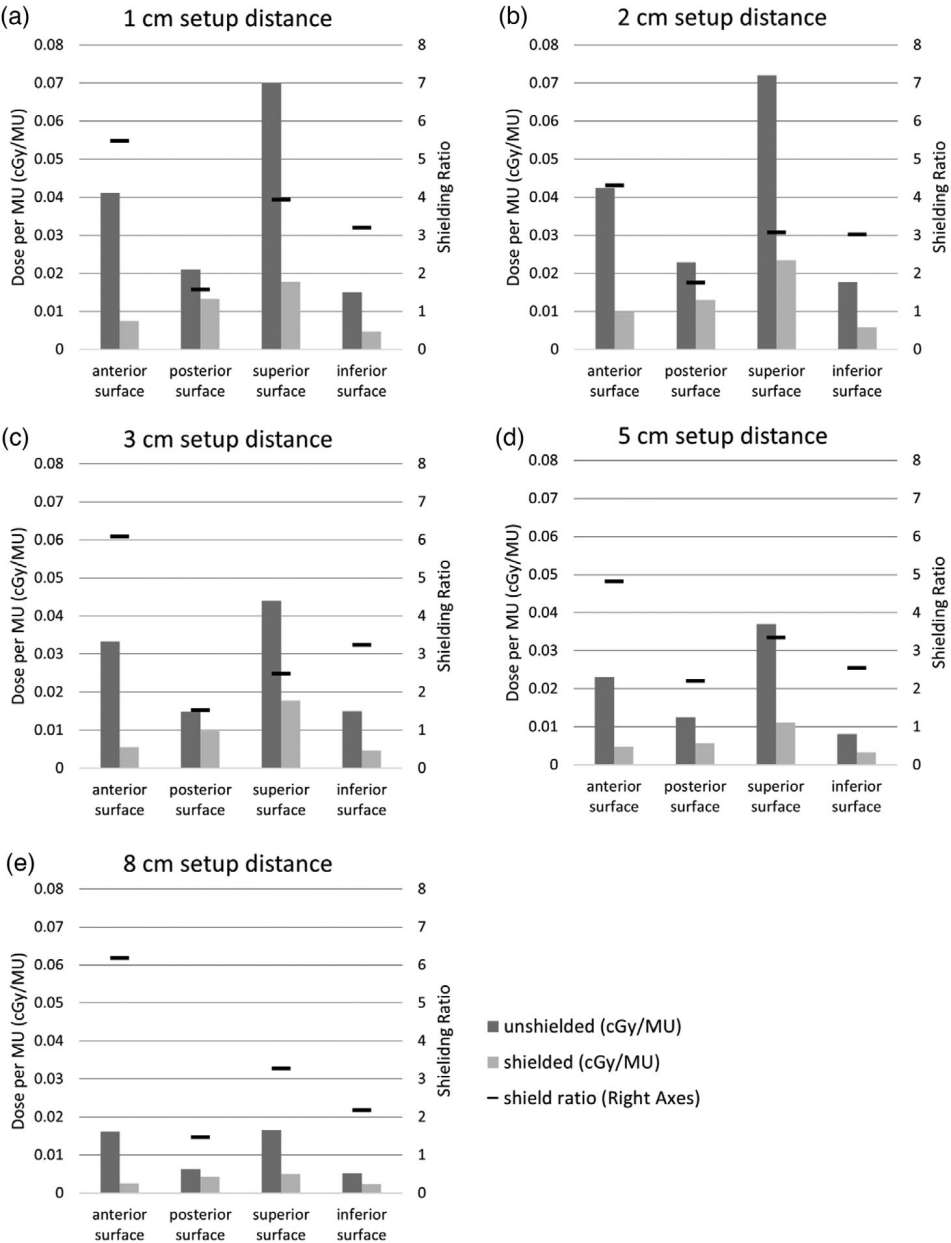
Dose‐to‐MU (bars) and shielding ratio (line, right axis) at surface locations on the scrotal phantom measured at different field‐to‐phantom distances.

A spatial variation of surface dose shielding ratio was also observed. As illustrated in Figure 2, anterior surfaces displayed the highest shielding ratios while the lowest ratios were measured at posterior surfaces. This pattern was consistent across all field‐to‐phantom distances. No systematic trend was observed between location‐averaged surface shielding ratio and field‐to‐phantom distance (Table 1, Figure S2), and this ratio was measured approximately 3 to 3.5 (average of 3.3).

**TABLE 1:** Location‐averaged shielding ratios of surface and internal doses at different field‐to‐phantom distances.

	1 cm	2 cm	3 cm	5 cm	8 cm
Surface measurement	3.54	3.04	3.33	3.22	3.27
Internal measurement	3.44	2.64	2.55	2.57	2.35

### 3.2 Internal dose variation with distance

In contrast to trends observed at the surface, minimal variation of the internal dose‐to‐MU was observed across different measurement locations inside the scrotal phantom. Figure 3 shows the internal dose‐to‐MU measured at different field‐to‐phantom distances, with and without the testicular shield. Internal dose‐to‐MU values measured in axial planes at the two representative setup distances (1 and 2 cm) were within 10% of the values measured at those distances in the coronal planes. Like the surface measurements, the location‐averaged internal dose‐to‐MU values also tended to decrease as the field‐to‐phantom distances increased for both shielded measurements (0.0072 to 0.0030 cGy/MU) and unshielded measurements (0.023 to 0.007 cGy/MU).

**FIGURE 3:**
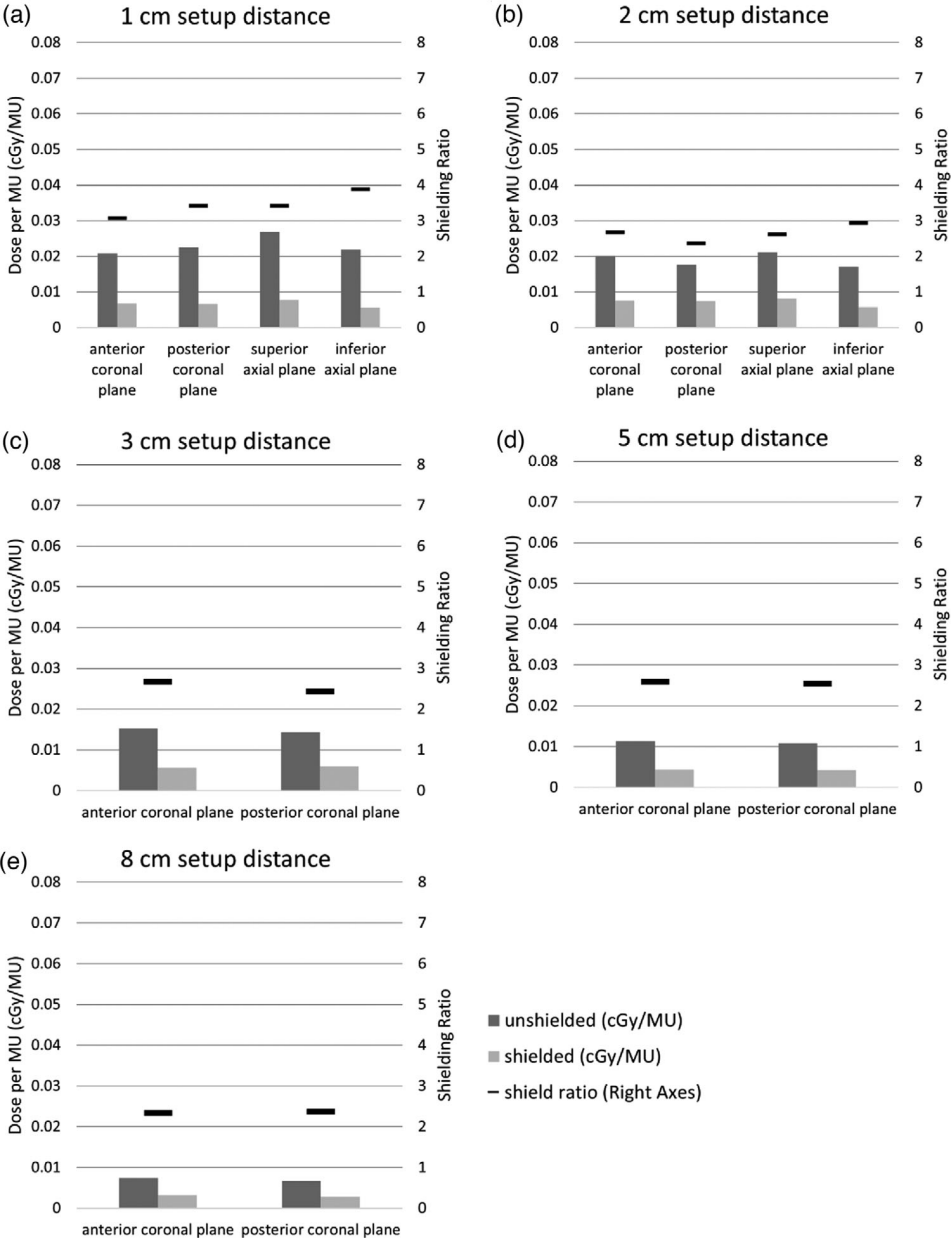
Dose‐to‐MU (bars) and shielding ratio (line, right axis) over internal planes within the scrotal phantom measured at different field‐to‐phantom distances.

As shown in Figure 3, consistent shielding ratios were measured at different locations inside the scrotal phantom, and no systematic trend was observed between the shielding ratio and field‐to‐phantom distance. The location‐averaged internal shielding ratios were lower than the surface shielding ratios at all field‐to‐phantom distances, ranging from 2.3 to 3.4 with an average of 2.7 (Table 1, Figure S2); however, the difference between the shielding ratios was not statistically significant according to the Wilcoxon signed‐ranks test with setup distance as the pairing variable (*N* = 5, *p* > 0.05).

### 3.3 VMAT dose and variation with energy

Figure 4 and Table 2 summarize the dose‐to‐MU measured for the 6 and 15 MV VMAT plans at different measurement locations on the scrotal phantom. Spatial variation of the surface dose‐to‐MU values was observed for both shielded and unshielded measurements. In both VMAT plans, superior surfaces exhibited the highest dose‐to‐MU value while the lowest values were measured at inferior surfaces. The 15 MV plan displayed slightly higher surface dose‐to‐MU measurements than the 6 MV plan, although only at the superior surface was the difference large enough to be potentially clinically meaningful (0.0051 vs. 0.0031 cGy/MU). In contrast, minimal spatial variation was observed in the internal dose measurements (Figure 4), and the dose‐to‐MU values were consistent between the two plans.

**FIGURE 4:**
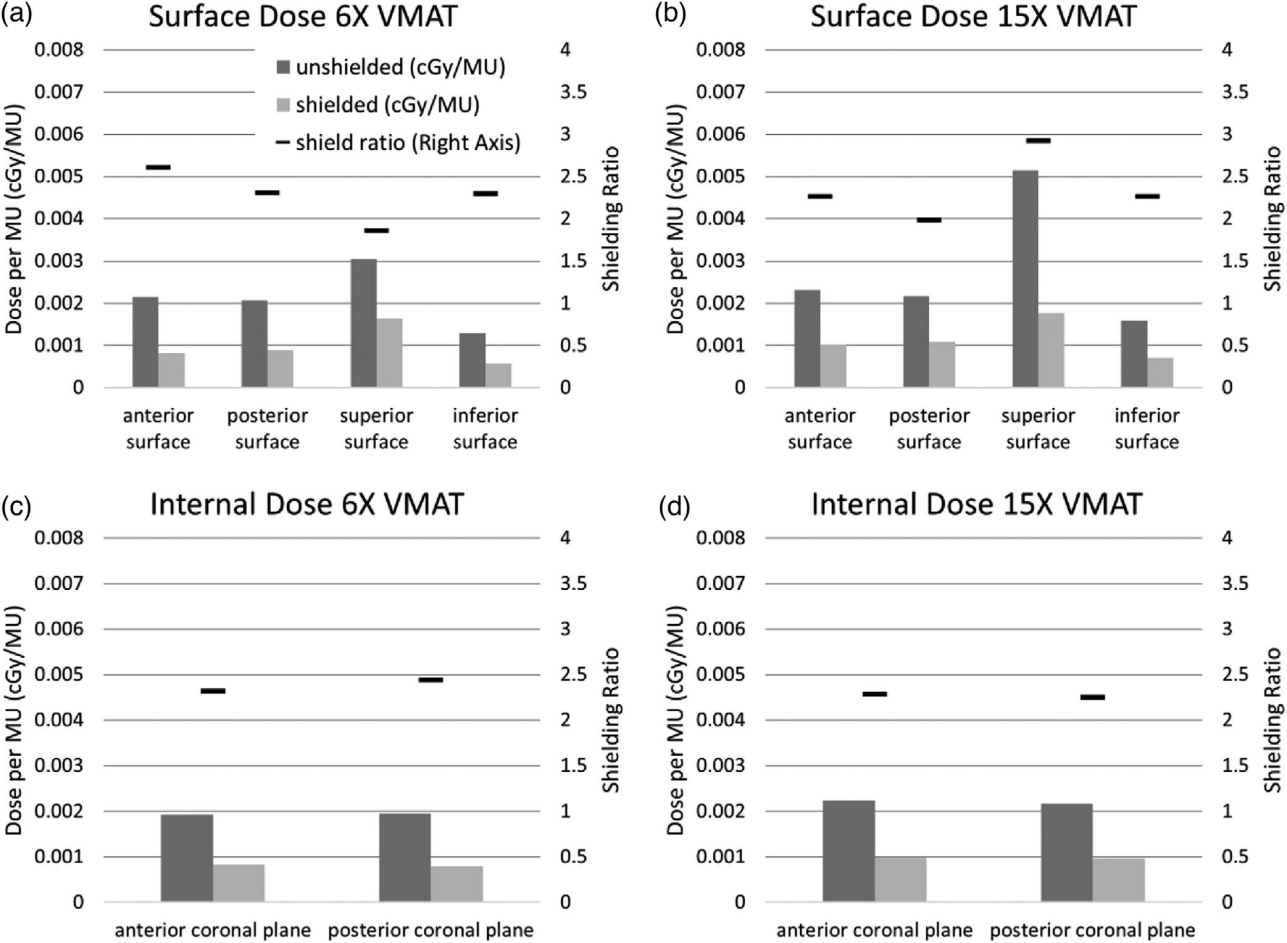
Dose‐to‐MU (bars) and shielding ratio (line) for surface and internal dose measurements, delivered with both 6 MV and 15 MV VMAT plans.

**TABLE 2:** Location‐averaged dose‐to‐MU and shielding ratios for 6 MV and 15 MV VMAT plans.

	6 MV		15 MV	
	Surface	Internal	Surface	Internal
Unshielded dose‐to‐MU (cGy/MU)	0.0021	0.0019	0.0028	0.0022
Shielded dose‐to‐MU (cGy/MU)	0.0010	0.0008	0.0011	0.0010
Shielding ratio	2.26	2.38	2.36	2.27

The location‐averaged surface shielding ratios were 2.26 for the 6MV plan and 2.36 for the 15 MV plan, whereas the shielding ratios for internal dose measurements were 2.38 and 2.27 for 6 MV and 15 MV, respectively. No consistent spatial variation of shielding ratios was observed between measurement locations, in contrast to the static beam shielding ratios. The overall shielding ratio (averaged across surface and internal measurements) was 2.30 for the 6 MV plan and 2.33 for the 15 MV plan, with no statistically significant difference (paired by measurement location, *N* = 6, *p* > 0.05).

## 4 DISCUSSION

In this study, scattered radiation dose to both the scrotal surface and internal regions was quantified during pelvic irradiation, and the effectiveness of a commercial testicular shield was evaluated for stationary‐beam and VMAT techniques. Under stationary gantry irradiation, surface dose‐to‐MU values varied among measurement locations on the scrotal phantom, whereas internal dose‐to‐MU values were more uniform and generally smaller. Across all field‐to‐phantom distances, the superior surface consistently exhibited the highest dose‐to‐MU values for both shielded and unshielded irradiations. This behavior is consistent with the measurement setup, as the superior surface is closest to the irradiated volume of the pelvis phantom, which serves as the dominant source of scattered radiation. In addition, the anterior surface displayed high unshielded dose‐to‐MU values, but more typical values when the testicular shield was present, resulting in comparatively large shielding ratios at this location. This observation suggests that the dose at the anterior surface is dominated primarily by externally scattered radiation, which can be effectively attenuated by the shield. In contrast, internal dose averages showed less spatial variation. While the average dose‐to‐MU values across internal measurement planes were quite similar, there were noticeable dose gradients within each plane, for instance, the coronal planes displayed higher doses at points of the phantom nearer to the field. If internal dose‐to‐MU was measured at other planes, especially planes closer to the phantom surface, the mean dose‐to‐MU would probably vary more with measurement location. The dose‐to‐MU, averaged across both surface and internal locations, trended downward with increasing field‐to‐phantom distances.

As with the surface dose‐to‐MU behavior, the surface shielding ratios also varied among locations, where the highest ratios ranged from 4.3 to 6.2 at the anterior surface while the lowest ranged from 1.5 to 2.2 at the posterior surface. Such behavior reflects variations in the relative contributions of externally scattered radiation and internal scatter. Locations dominated by external scatters in unshielded conditions showed higher shielding ratios, whereas locations with a larger relative contribution from internally scattered radiation showed lower shielding ratios. The internal shielding ratios measured at different coronal planes were comparable and remained consistent across different field‐to‐phantom distances. For all the field‐to‐phantom distances, the location‐averaged surface shielding ratio ranged approximately from 3 to 3.5, while the location‐averaged internal shielding ratio ranged about 2.4 to 3.4. No systematic dependence of shielding ratio on field‐to‐phantom distance was observed.

Photon energy had limited influence on the scattered dose from VMAT deliveries measured at the scrotal phantom. Consistent location‐averaged surface dose‐to‐MU values were observed between the two energies for both shielded and unshielded measurements. Only one location displayed a large change in dose‐to‐MU with energy: the superior surface under unshielded conditions. This may be because the superior surface was in range of higher energy electrons liberated by the 15 MV beam which contributed to the unshielded measurement. In addition, location‐averaged internal dose‐to‐MU values were nearly identical between the two energies for both shielded and unshielded measurements, indicating that photon energy has minimal influence on the scattered radiation reaching deeper regions of the phantoms. The VMAT plans measured substantially lower dose‐to‐MU values compared with the stationary gantry irradiation, by one order of magnitude at surface locations and by about sevenfold at internal locations, reflecting differences between the two delivery techniques.

The VMAT measurements showed shielding ratios of approximately 2.3 throughout the surface and internal volume of the scrotal phantom for both low and high photon energies. Taken together with the lack of relationship between shielding ratio and setup distance discussed above, the results suggest that the 6 cm Pb testicular shield with 1.27 cm wall thickness can provide a dose reduction factor of 2 to 3 to the testes during delivery of a typical VMAT plan, with limited dependence on photon energy or out‐of‐field distance. When translated to a hypothetical rectal VMAT treatment of 1000 MU per fraction under conventional fractionation of 50.4 Gy in 28 fractions, the average dose‐to‐MU value would correspond to an estimated scrotal dose of approximately 2 cGy per fraction, or roughly 58 cGy over a treatment course, without testicular shielding. This cumulative dose would be reduced to approximately 25 cGy with the shield in place. On the other hand, when treatments are delivered with a stationary gantry or limited number of beam angles, shielding effectiveness of this shield may vary more substantially depending on beam direction relative to the testes and shield, with dose reduction factors ranging from 1.5 to 6 over the interior volume of the shield. For a palliative pelvic treatment with anterior‐posterior parallel beams delivered in one fraction of 8 Gy with 900 MU, the estimated overall testicle dose at 3 cm distance would be approximately 21 cGy without the shield and would average 7 cGy with the shield. These estimates, however, may vary depending on actual field arrangements, plan modulation, and target‐to‐testes distance in individual patients.

The photoneutron production should be considered in the interpretation of the 15 MV results. The EBT3 film used in this study is insensitive to neutrons and primarily responds to photon radiation, including secondary photons generated through neutron interactions. Consequently, the measured shielding ratios for the 15 MV VMAT plan represented photon dose reduction and did not directly quantify the neutron dose contribution. If photoneutron production substantially contributed to the out‐of‐field dose, differences in shielding effectiveness may not be fully reflected by radiochromic film measurements alone. In this study, comparable shielding ratios were observed for both energies, suggesting that neutron induced secondary photons had limited impact on the measured photon dose under the measurement conditions investigated. Nevertheless, neutron dose should be considered when evaluating testicular shielding for high energy photons due to the high relative biological effectiveness of neutrons and the limited attenuation of neutrons by Pb.

Our data are in good agreement with those of Jia, et al., who found average shielding ratios of 2.18 from Monte Carlo simulation and 2.28 from in vivo measurements using TLDs.^10^ Other work has measured doses corresponding to shielding ratios similar to those measured in this paper (Table S1), including ratios of 3 to 4.375 measured in phantom with an ion chamber over multiple studies, ratios of approximately 3 for comparable thickness shields derived from Monte Carlo simulation, and ratios of between 1 and 2 measured in various in vivo setups.^11^, ^12^, ^13^, ^14^ Some investigators found doses corresponding to ratios rising to the order of 10 in phantoms and in vivo, likely due to the use of different radiation machines, fields, setups, detectors, and/or shielding types.^7^, ^9^, ^15^ One study assessed testicular surface dose using a phantom and radiochromic film setup very similar to ours and found an almost negligible shielding dose reduction of only 10%. However, this study delivered only one fraction of radiation per film measurement, which corresponded to prescription doses of 200 cGy for their posterior‐anterior field or 167 cGy for their dogleg field.^8^ Our study, therefore, benefits from substantially higher signal to noise ratios, which are particularly important when measuring dose with radiochromic film.

This study did not find a relationship between shielding ratio and setup distance, in agreement with Ravichandran, et al., who found similar ratios at 3 and 7.5 cm, but in contrast to other studies that did find a relationship.^9^, ^14^, ^15^ However, Singhal's study was performed using cobalt teletherapy units, and Fraass’ work utilized a MeVatron XII and a Clinac 18. These machines are likely to have very different distributions of head leakage relative to internal scatter than modern treatment machines like the Varian Truebeams used in our study. If the relative contributions of head leakage and internal scatter from these machines varied strongly with distance from the field edge in the 1–8 cm range, then this might explain the relationship they observed. The study of Park, et al. also directly compared 6 to 15 MV shielding ratios and reported higher shielding efficacy at 15 MV than 6 MV. However the magnitude of the difference was quite small when the shield thickness simulated was similar to ours.^13^

In addition to the softer energy spectrum of out‐of‐field scatter relative to the 6 MV calibration beam, Pb testicular shielding generates characteristic Pb fluorescence x‐rays, predominantly K‐shell x‐rays (approximately 72 to 88 keV). Although the response of EBT3 film has not been directly investigated in this energy range, studies showed that the relative sensitivity of modern EBT3 films is generally within 5% from about 40 keV to MeV photon energies.^17^, ^19^, ^20^ Therefore, Pb K‐shell x‐rays may introduce additional systematic uncertainty into dose measurements beneath and adjacent to the shield. In addition, because each experimental condition was evaluated using a single film irradiation, film‐to‐film reproducibility and repeat‐measurement uncertainty were not assessed. Future studies incorporating replicate measurements would allow a more comprehensive characterization of measurement uncertainty.

One limitation of this study is that the measurements were performed using a simplified phantom geometry, which enhances setup reproducibility, but does not represent actual anatomy or inter‐fraction setup variation. The size of the phantom also represents an overestimate of attenuation within the volume of the testicular shielding because, in a clinical setup, most of that volume will be filled with air instead of plastic. In addition, the PLA phantom was less dense than soft tissue (approximately ‐150 HU), which may underestimate photon attenuation and backscatter relative to clinical situation. A more accurate facsimile of the shielded anatomy, with respect to shape and density, may allow for more accurate estimates of absorbed testicular dose. Surface dose measurements were measured as point doses in this study, but different film placement geometry or digital dose calculations could be used to further characterize the three‐dimensional dose distributions within the shielding. Another limitation is that neutron dose associated with 15 MV photon beams was not directly evaluated in this study. Further studies incorporating dedicated neutron‐sensitive detectors are warranted to quantify the neutron dose contribution and provide a more comprehensive evaluation of the shielding performance of the Pb testicular shield during high energy photon treatments. Other future work could compare our phantom measurement results with in vivo assessments of shielding efficacy in similar conditions and assess the shielding ratios of other commercially available testicular shielding.

## 5 CONCLUSION

A testicular shield with a lead wall thickness of 1.27 cm can effectively reduce surface and internal testicle dose by a factor of approximately 2 to 3. This shielding ratio remains substantially similar across a variety of setup conditions in VMAT and static gantry irradiations and could therefore serve as a valuable clinical rule of thumb. However, neutron dose should be considered when using high energy photon beams. These results provide practical guidance for estimating testicle doses and the potential benefit of shielding in radiotherapy treatment of sites near the testes.

## AUTHOR CONTRIBUTIONS

Joseph Speth designed and performed measurements, analyzed data, and was a major contributor in writing the manuscript. Josh Kilian‐Meneghin helped initiate the project, collected preliminary data, and revised the manuscript. Salma K. Jabbour helped initiate the project and revised the manuscript. Yin Zhang, Xiao Wang, Zhenyu Xiong, Ke Nie, and Ning J Yue helped design measurements and revised the manuscript. Xinxin Zhang helped initiate the project, designed and performed measurements, and was a major contributor in writing the manuscript. All authors read and approved the final manuscript.

## CONFLICT OF INTEREST STATEMENT

The authors declare no conflicts of interest.

## Supporting information



Supporting Information

Supporting Information

## Data Availability

Authors will share data upon request to the corresponding author.
