# Supplementary material for: Evaluation of testicular shielding effectiveness for surface and internal photon dose using a 3D‐printed scrotal phantom
Source: J Appl Clin Med Phys. 2026 Oct 5;27(10):e70842. doi: 10.1002/acm2.70842 (PMC13639242; doi:10.1002/acm2.70842)
Supplement: Supplementary file 2 [file ACM2-27-e70842-s002.docx]

Supplementary Material:

**S1. Literature Review**

| Supplemental Table 1: Conditions and results of previous shielding studies | | | | | | |
| --- | --- | --- | --- | --- | --- | --- |
| Author | Year | Beam Energy | Setup Distance | Type of Shield | Type of Detector/  simulation | Results |
| Lieng, et al. | 2018 | 18 MV | Median 13 cm | 1.25 cm thick lead | MOSFET | Mean dose 2.6 cGy with shielding, 28.6 cGy without |
| Fricker, et al. | 2013 | 18 MV | Not stated, dependent on technique | Custom, lead, thickness not stated | EBT2 Film | Paraaortic technique: 30 cGy with shielding, 31 cGy without  Dogleg technique: 61 cGy with shielding, 68 cGy without |
| Fraass, et al. | 1985 | 10 MV | Up to 30 cm | Custom, lead, >3 cm thick top and bottom, 1 cm sides | TLD | Shielding ratios of 3-10X, dropping with increasing distance |
| Jia, et al. | 2023 | 15 MV | Approx. 3cm | 15.6 mm thick lead | TLD and Geant4 | Simulation shielding ratio of 2.18, *in vivo* shielding ratio of 2.28 |
| Nazmy, et al. | 2007 | 6 MV | External shielding at field edge | External shield, 5 HVL of Cerrobend | diodes | 22% decrease in testicular dose with shielding |
| Park, et al. | 2025 | 6 MV and 15 MV | Variable in simulation, 0-15 cm | Variable thickness in simulation, 1.5 cm lead *in vivo* | Geant4 and OSLD | 50-75% dose reduction, depending on thickness in simulation. Less dose reduction *in vivo* |
| Ravichandran, et al. | 2008 | 15 MV | 3 and 7.5 cm | 1.5 cm Cerrobend | Ion chamber and TLD | Shielding ratios of 4.375 at 7.5 cm and 4.176 at 3 cm |
| Singhal, et al. | 2014 | Co-60 | 3.5 and 8 cm | 2 cm Cerrobend | Ion chamber and TLD | Shielding ratios of 7.22 at 3.5 cm and 10.33 at 8cm |

**S2. Film Calibration**

The Gafchromic EBT3 film was calibrated with solid water phantom using a 6 MV photon beam for a dose range of 0-10 Gy. Films were scanned 24 hours post-irradiation using an Epson 11000XL flatbed scanner (Seiko Epson Corp., Suwa, Nagano, Japan) at 300 dpi in 48-bit color with color corrections disabled. Polynomial regression fits were applied in both the low dose range (0 to 3 Gy) and high dose range (3 to 10 Gy) to characterize the relationship between film red channel pixel value and absorbed dose. Films obtained from the phantom measurements were scanned and analyzed under identical conditions as the calibration films.

Supplemental Figure 1 shows the film calibration curves acquired per our institutional calibration protocol. Absolute doses among our measured data ranged from 28 cGy to 586 cGy, depending on setup and number of MU delivered.

Supplemental Figure 1. Film calibration curves for dose range of 0-3 Gy and 3-10 Gy. The relative pixel value was the difference of the background reading and the measured signal of each irradiated film.

**S3. Additional Results**

The small increase in dose-to-MU at 2cm setup distance relative to 1cm setup distance, in some setup conditions, was not statistically significant when 1cm values were paired with 2cm values by measurement location (N = 6, p-value > 0.05)) and the Wilcoxon signed-ranks test was run separately for shielded and unshielded conditions (Supplemental Figure 2). The general downward trend is consistent with reduced scatter contribution as the separation between the treatment field and the scrotal phantom increases.

Supplemental Figure 2. Location-averaged surface and internal dose-to-MU, with and without testicle shields, at different field-to-phantom distances.
